# Neurosyphilis Diagnosis and Treatment in Psychiatric Hospitals: A Retrospective Study

**DOI:** 10.31083/AP38794

**Published:** 2025-02-28

**Authors:** Yajie Wang, Zhili Hu, Heping Zheng, Xiaolin Qin, Changchang Li, Hui Zhang, Lianhong Zheng, Wujian Ke

**Affiliations:** ^1^Dermatology Hospital, Southern Medical University, 510091 Guangzhou, Guangdong, China; ^2^Department of Sexually Transmitted Infection, Guangdong Provincial Center for Skin Diseases and STDs Control, 510091 Guangzhou, Guangdong, China; ^3^Laboratory of Sexually Transmitted Infection, Guangzhou Key Laboratory for Sexually transmitted Diseases Control, 510091 Guangzhou, Guangdong, China; ^4^Department of Neurology, Affiliated Hospital, Hebei Engineerning University, 056000 Handan, Hebei, China

**Keywords:** neurosyphilis, psychiatric disorders, psychiatric hospital

## Abstract

**Background:**

Neurosyphilis, caused by *Treponema pallidum* invading the nervous system, can lead to severe neurological complications across all stages of syphilis. Misdiagnosis is common, exacerbated by overlapping psychiatric conditions and diagnostic limitations. This study aims to improve the understanding and management of neurosyphilis in psychiatric settings to enhance diagnostic accuracy and treatment efficacy.

**Methods:**

A retrospective study used data from the Chinese Case Report System (CCRS) spanning 2014–2018. Four municipal psychiatric hospitals in Guangdong were chosen based on syphilis rates and psychiatric patient volumes. Sociodemographic data, syphilis history, symptoms, diagnostic and treatment details, and laboratory results were reviewed. The Brief Psychiatric Rating Scale (BPRS) assessed psychopathology symptoms. Treatment efficacy was evaluated using BPRS scores before and after standard treatment. Diagnoses followed national guidelines, with statistical analyses performed using logistic regression and *t*-tests.

**Results:**

Out of 69,436 psychosis patients screened, 1588 were diagnosed with syphilis, with 262 (16.5%) of these cases identified as neurosyphilis. Syphilis prevalence mildly declined from 2.8% (2014) to 2.0% (2016), while neurosyphilis cases increased marginally from 0.34% (2014) to 0.39% (2018). Confirmatory Cerebrospinal Fluid (CSF) tests were conducted in only 30.2% of neurosyphilis cases. Standard therapy was administered to 66.8% of patients, with significantly better outcomes in confirmed cases (*p* < 0.001).

**Conclusions:**

Diagnosing and treating neurosyphilis in Guangdong’s psychiatric hospitals remains challenging. Accurate diagnosis and standardized treatment protocols are essential to effectively manage both syphilis and associated mental health complications caused by neurosyphilis.

## Main Points

1. Prevalence and Diagnosis: Among 1588 syphilis cases, 262 (16.5%) were reported as neurosyphilis. Only 30.2% (79) of these cases were confirmed through CSF tests.

2. Psychiatric Symptoms: 34.7% of neurosyphilis cases were initially diagnosed with schizophrenia, highlighting the challenge of differential diagnosis in psychiatric settings.

3. Standard Treatment: Of the reported neurosyphilis cases, 66.8% received standard treatment. Confirmed cases had a significantly higher rate of standard treatment compared to suspected cases.

4. Treatment Efficacy: BPRS scores indicated significant improvement in psychotic symptoms after standard treatment, particularly in confirmed neurosyphilis cases.

5. Diagnostic Challenges: The study underscores the difficulty in diagnosing neurosyphilis due to varied clinical manifestations and the need for improved diagnostic capabilities, especially in lower-grade hospitals. MRI plays a crucial role in diagnosing neurosyphilis, detecting various manifestations and brain abnormalities that often precede symptoms, emphasizing its importance in early detection.

## 1. Introduction

Syphilis is a resurgent sexually transmitted disease worldwide, with 6.3 million new syphilis cases each year among people aged 15–49 [1, 2]. According to the American Centers for Disease Control and Prevention (CDC), 176,713 syphilis cases were reported in 2021. The rate of primary and secondary syphilis increased by 28.6% from 2020 to 2021 [3]. In China, 535,819 cases of syphilis (all stages) were reported, with the incidence rising from 30.93 to 38.37 cases per 100,000. This reflects an average annual growth rate of 4.41% during 2014–2019 [4]. Guangdong Province in South China is experiencing a syphilis epidemic. In 2019, 6276 cases were reported in Guangdong, accounting for 11.7% of national cases [5].

*Treponema pallidum* (*T. pallidum*), the causative agent of syphilis, can spread to the central nervous system hours to days after inoculation, leading to neurosyphilis at any stage of syphilis [6]. If untreated, 4%–10% of patients with syphilis develop neurosyphilis [7]. Neurosyphilis can cause severe neurological damage, manifesting as acute meningitis, cranial nerve abnormalities, or stroke due to central nervous system (CNS) arteritis, and may progress to chronic conditions like general paresis or tabes dorsalis, leading to dementia, motor dysfunction, and sensory loss. It can also affect the eyes and ears, resulting in visual impairment, hearing loss, or balance issues [8]. However, the true incidence of neurosyphilis is hard to determine due to frequent misdiagnoses, a lack of accurate microbiological tests, varied disease symptoms, and different clinical diagnostic criteria [9]. Between 2009 and 2021, 7486 cases of neurosyphilis were reported in China [10]. Insufficient detection of cerebrospinal fluid and the low sensitivity (30–50%) of non-specific antibody tests complicate diagnosis and treatment. Additionally, the standard 10–14 day hospital stay required for neurosyphilis treatment is often affected by hospital conditions, patient financial constraints, and preferences to avoid prolonged hospitalization, resulting in non-standard treatment. Our early cross-sectional study found that 48.3% of cases were misdiagnosed among 3805 patients with neurosyphilis and tertiary syphilis reported during 2009–2014, and only 27.1% of the correctly diagnosed cases of late neurosyphilis received standard treatment [11]. The diagnosis of neurosyphilis is generally difficult [12].

Numerous psychotic symptoms are caused by neurosyphilis, which can inform a differential diagnosis of patients with psychosis [7]. Recently, the incidence of syphilis, especially neurosyphilis, has increased [13]. This study aims to enhance the diagnosis and treatment of neurosyphilis in psychiatric hospitals. Given the frequent misdiagnosis resulting from overlapping psychiatric symptoms and a rising incidence of neurosyphilis, this research evaluates diagnostic and management practices in four municipal psychiatric hospitals in Guangdong Province from 2014 to 2018. The goal is to improve patient outcomes and reduce the overall impact of neurosyphilis.

## 2. Materials and Methods

### 2.1 Study Design and Data Collection

All records of confirmed syphilis cases reported to the Chinese Case Report System (CCRS) between 2014 and 2018 at the Guangdong Provincial Center for Skin Diseases and Sexual Transmitted Diseases (STDs) control were identified. Four municipals psychiatric hospitals in Guangdong Province were selected based on syphilis rates and the number of inpatients with mental illness. Guangzhou and Foshan psychiatric hospitals are Grade III hospitals. Jiangmen and Shaoguan hospitals are Grade II hospitals. In the Chinese health system, hospital grades are based on their functions, facilities, and technical capabilities, ranging from tertiary to primary. The inclusion criteria for this study encompass psychiatric patients who visited our hospital during the period spanning from 2014 to 2018. Specifically, among these patients, individuals with positive syphilis serology results were included in the research. Conversely, those who tested negative for syphilis serology were excluded from the analysis. A total of 69,436 patients with psychosis were screened for syphilis by serological tests in these four hospitals. A total of 1588 patients were diagnosed with syphilis, and 262 were reported as having neurosyphilis. We collected information on sociodemographic characteristics, syphilis history, clinical symptoms, diagnosis status, treatment status, and laboratory test results for all syphilis cases. This study was approved by the Institutional Ethics Committee of Southern Medical University Dermatology Hospital (GDDHLS-20181202). Since our study is based on a retrospective analysis of data retrieved from a historical database, all data used were de-identified to ensure privacy and comply with ethical standards, therefore, obtaining informed consent from participants was not applicable or necessary for this research.

### 2.2 Evaluation of Psychiatric Symptoms

The brief psychiatric rating scale (BPRS) was used to systematically assess signs and symptoms of psychopathology [14, 15]. The BPRS consisted of 18 symptom constructs, each rated on a seven-point (0 to 6) scale of severity ranging from “not present” to “extremely severe”. The attending psychiatrists rated the manifested psychopathology of patients using BPRS and provided a global clinical severity rating on a 7-point scale before and after standard treatment.

### 2.3 Diagnosis and Treatment

The definition of syphilis and neurosyphilis cases followed the diagnostic criteria of the Guidelines for Diagnosis and Treatment of STDs published by the National Center for STDs Control [16], similar to the criteria of STDs Treatment Guidelines of US CDC, 2015 [17]. For syphilis diagnosis, the serum rapid plasma reagin test was used for screening, followed by the Treponema pallidum particle agglutination (TPPA) test for confirmation. Suspected neurosyphilis was defined by (1) history of primary, secondary, or latent syphilis history; and (2) clinical manifestations involving central nervous system. Confirmed cases also required laboratory confirmation with either a reactive Cerebrospinal Fluid-Venereal Disease Research Laboratory (CSF-VDRL) test or a CSF white blood cell count of >20 cells/uL [12]. Standard treatment included aqueous crystalline penicillin G 18–24 million units per day, administered as 3–4 million units intravenous injection (IV) every 4 h or continuous infusion for 10–14 days, or procaine penicillin G 2.4 million units intramuscular injection (IM) once daily plus probenecid 500 mg orally 4 times/day, both for 10–14 days. Alternative ceftriaxone, doxycycline or erythrocin were used if the patient was allergic to penicillin, as suggested by the Guidelines for Diagnosis and Treatment of Sexual Transmitted Diseases [16].

### 2.4 Statistical Analysis

Descriptive statistics for sociodemographic characteristics were reported for neurosyphilis cases (confirmed and suspected cases). The annual prevalence rates of syphilis and neurosyphilis from 2014 to 2018 were calculated, and the trend of annual rate was analyzed using linear regression. Logistic regression modeling was applied to estimate the odds ratio and corresponding 95% Confidence Intervals (CIs) for associated factors on diagnosis with “suspected” neurosyphilis cases. The *t*-test was used to compare BPRS scores before and after treatment to evaluate changes in the mental symptoms of neurosyphilis. All tests were two-sided with a type 1 error level of 0.05, and all analyses were conducted using IBM SPSS Statistics 20.0 software (IBM, Armonk, NY, USA).

## 3. Results

### 3.1 Characteristics of Neurosyphilis Cases Reported

A total of 69,436 patients with psychosis were screened for syphilis by serological tests in four municipal psychiatric hospitals from 2014 to 2018. Out of these, 1588 patients were diagnosed with syphilis, of whom 262 (16.5%) were reported as neurosyphilis. The average age of neurosyphilis cases was 58.8 years (standard deviation (SD) = 13.1), and 77.9% of the cases were male. Of the neurosyphilis cases, 79 (30.2%) were confirmed, while 183 (69.8%) were suspected. The majority of neurosyphilis cases (34.7%) were initially diagnosed with schizophrenia, followed by organic (19.1%), bipolar (16.8%), and paranoid (14.1%) disorders upon their first visit to the psychiatric hospitals. More neurosyphilis cases (56.5%) were reported in the two Grade II hospitals than the Grade III hospitals (Table 1).

**Table 1.** **Demographics of reported neurosyphilis cases in four municipal psychiatric hospitals during 2014–2018 (n = 262)**.

Variable		Confirmed cases (N = 79)		Suspected cases (N = 183)		Overall (N = 262)		*p* value
		Number	Percent (95% CI)	Number	Percent (95% CI)	Number	Percent (95% CI)	
Gender	Male	62	78.5 (69.2, 87.7)	142	77.6 (71.5, 83.7)	204	77.9 (72.8, 82.9)	0.874
	Female	17	21.5 (12.3, 30.8)	41	22.4 (16.3, 28.5)	58	22.1 (17.1, 21.2)	
Age	<45 y	19	33.3 (20.7, 46.0)	38	29.3 (23.0, 35.3)	57	30.2 (24.6, 35.7)	0.554
	≥45 y	60	66.7 (54.0, 79.3)	145	70.7 (64.5, 77)	205	69.8 (64.3, 75.4)	
Mental symptom on first visit	Schizophrenia	25	31.6 (21.1, 42.1)	66	36.1 (29.0, 43.1)	91	34.7 (28.9, 40.5)	0.377
	Organic disorder	19	24.1 (14.4, 33.7)	31	16.9 (11.5, 22.4)	50	19.1 (14.3, 23.9)	
	Bipolar disorder	14	17.7 (9.1, 26.3)	30	16.4 (11.0, 21.8)	44	16.8 (12.2, 21.4)	
	Paranoid disorder	13	16.5 (8.1, 24.8)	24	13.1 (8.2, 18.1)	37	14.1 (9.90, 18.4)	
	Other	8	10.1 (3.3, 16.9)	32	17.5 (11.9, 23.0)	40	15.3 (10.9, 19.7)	
Year	2014	11	13.9 (6.1, 21.7)	31	16.9 (11.5, 22.4)	42	16.0 (11.6, 20.5)	0.983
	2015	13	16.5 (8.1, 24.8)	30	16.4 (11.0, 21.8)	43	16.4 (11.9, 20.9)	
	2016	16	20.3 (11.2, 29.3)	36	19.7 (13.9, 25.5)	52	19.8 (15.0, 24.7)	
	2017	18	22.8 (13.3, 32.2)	40	21.9 (15.8, 27.9)	58	22.1 (17.1, 27.2)	
	2018	21	26.6 (16.6, 36.5)	46	25.1 (18.8, 31.5)	67	25.6 (20.3, 30.9)	
Hospital tier	Grade III	47	59.5 (48.4, 70.6)	67	36.6 (29.6, 43.7)	114	43.5 (37.5, 49.6)	<0.001
	Grade II	32	40.5 (29.4, 51.6)	116	63.4 (56.3, 70.4)	148	56.5 (50.4, 62.5)	

CI, confidence interval.

### 3.2 Prevalence of Syphilis and Neurosyphilis in the Psychiatric Hospitals 

**Fig. 1.**
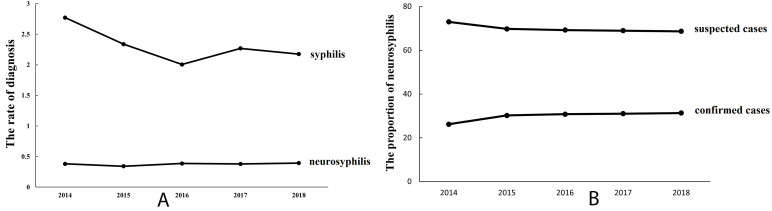
**Prevalence and Proportion of Syphilis and Neurosyphilis Cases in Four Psychiatric Hospitals (2014–2018)**. Prevalence of reported syphilis & neurosyphilis (A), and proportion of confirmed/suspected neurosyphilis cases (B) in 4 psychiatric hospitals, 2014–2018.

All 1588 syphilis cases reported from the four psychiatric hospitals were reviewed and confirmed according to the diagnostic criteria described above [18]. The annual prevalence rates of syphilis from 2014 to 2018 were 2.8% (305/11,000), 2.3% (294/12,578), 2.0% (270/13,462), 2.3% (348/15,343) and 2.2% (371/17,053), respectively, showing a significant decrease since 2014 to 2016 (*p* < 0.05). The rates of neurosyphilis cases reported increased slightly from 0.34% (43/12,284) in 2015 to 0.39% (67/17,053) in 2018 (Fig. 1A). Among 262 reported neurosyphilis cases, only 79 (30.2%) met the diagnostic criteria for neurosyphilis, whereas 183 (69.8%) were considered suspected neurosyphilis. The proportions of confirmed neurosyphilis cases were 26.2% (11/42), 30.2% (13/43), 30.8% (16/52), 31.0% (18/58), and 31.3% (21/69) from 2014 to 2018, showing a slight increase during the study period (Fig. 1B).

### 3.3 Diagnosis and Treatment According to the Guideline 

Out of 262 reported neurosyphilis cases, only 79 (30.2%) were diagnosed with neurosyphilis based on CSF tests. In the Grade II hospitals, 83 (71.6%) without CSF results were diagnosed as suspected neurosyphilis, compared to 42 (62.7%) in the Grade III hospitals. All 262 reported neurosyphilis cases received Anti-Treponema pallidum treatment, but only 175 (66.8%) cases had the standard therapy as per the guideline [18]. The rate of standard treatment in the confirmed neurosyphilis group was significantly higher than that in suspected cases (*p* < 0.001, Table 2). BPRS scores before and after treatment were compared, showing significantly higher score changes in confirmed cases compared to suspected cases (*p* < 0.001, Table 2).

**Table 2.** **Diagnosis and treatment of neurosyphilis**.

Variable		Confirmed cases (N = 79)	Suspected cases (N = 183)	*p* value
Diagnosis with lumbar puncture and CSF test (N = 137), n (%)	Grade III hospitals	47 (59.5)	25 (37.3)	0.03
	Grade II hospitals	32 (40.5)	33 (28.5)	
Treatment status, n (%)	Standard treatment	69 (87.3)	106 (57.9)	<0.001
	Non-standard treatment	10 (12.7)	77 (42.1)	
BPRS (mean ± (SD))	Before treatment	74.6 ± 12.7	71.1 ± 14.2	<0.001
	After treatment	45.3 ± 17.4	58.9 ± 14.7	

CSF, cerebrospinalfluid; BPRS, Brief Psychiatric Rating Scale; SD, standard deviation.

## 4. Discussion

Our study focused on neurosyphilis among syphilis cases attending the psychiatric hospital. Among all the syphilis patients screened, 16.5% were diagnosed with neurosyphilis, in which only one-third were confirmed and two-thirds were treated with standard therapy. The findings indicate that neurosyphilis might be frequently challenging in patients with mental disorders, complicating both diagnosis and treatment. Diagnosing and treating neurosyphilis is challenging due to inadequate cerebrospinal fluid testing in Grade II hospitals and the low sensitivity (30–50%) of non-specific antibody tests in Grade III hospitals. Complex clinical manifestations further complicate diagnosis. Treatment requires a 10–14-day hospital stay, which many Grade II hospitals cannot accommodate. Financial constraints and patient preferences to avoid extended hospitalization often result in incomplete or suboptimal care, worsening the condition’s management.

Psychotic symptoms resulting from neurosyphilis are varied and can contribute to differential diagnosis of many psychotic conditions [18, 19]. Rozwens *et al*. [19] reported that 27% of neurosyphilis patients exhibited psychiatric symptoms [20]. Similarly, among 149 HIV-negative Chinese patients with neurosyphilis, 46 out of 58 with general paresis presented with psychiatric presentations [20]. In this study, 34.7% neurosyphilis cases were diagnosed with schizophrenia, 19.1% organic, 16.8% bipolar, and 14.1% paranoid disorders on their first visit. Friedrich *et al*. [18] recommended routine screening tests in the psychiatric field. About 3% of prevalence rates of syphilis were reported among patients with psychosis [21]*.* We observed that the syphilis prevalence among patients with psychosis was 2.3% from 2014 to 2018, aligning with trends in Guangdong Province [22]. Most neurosyphilis cases involved males and individuals over 45 years old, mirroring the broader trend of increasing syphilis rates among older men, where the male-to-female ratio of syphilis was 1.8, with cases in men aged ≥50 years rising significantly from 13.8 per 100,000 in 2004 to 112.0 per 100,000 in 2019 [13].

Diagnosing neurosyphilis remains challenging because it can occur at any stage of syphilis infection and presents with diverse clinical manifestations, compounded by the lack of specific diagnostic tests. CSF examination is recommended in the presence of serologic positive of syphilis in the serum and a clinical syndrome that is consistent with neurosyphilis [17]. The US CDC defines confirmed neurosyphilis as (1) any syphilis stage and (2) a reactive CSF-VDRL [17]. Symptomatic neurosyphilis can present as meningitis, gumma, meningovascular involvement, brain parenchyma involvement, meningomyelitis, tabes dorsalis, or peripheral nervous system involvement. Magnetic resonance imaging (MRI) is crucial for diagnosing neurosyphilis. He C *et al*. [23] showed symptomatic neurosyphilis patients had more brain abnormalities on MRI, like infarcts and atrophy. These findings often precede symptoms, highlighting MRI’s importance in early detection. MRI is vital for diagnosing general paresis, a common neurosyphilis, by revealing brain changes despite a lack of definitive diagnosis and non-specific symptoms. It aids in timely and accurate diagnoses [24]. MRI’s advanced imaging technique, high-resolution vessel wall imaging (HR-VWI), accurately diagnosed neurosyphilis-related vascular complications, confirmed the cause, and monitored treatment [25]. However, in our study, out of 262 cases reported as neurosyphilis, 137 underwent lumbar puncture and CSF testing. The testing rates were significantly higher in Grade III hospitals (63.2%) compared to Grade II hospitals (43.9%), respectively (*p* < 0.001). To uphold evidence-based medicine principles, it is crucial to emphasize discussions on valid cases of neurosyphilis supported by comprehensive diagnostic evaluations, including CSF testing where indicated. Improving adherence to diagnostic guidelines across hospital grades, enhancing healthcare provider awareness, and advocating for standardized testing protocols are essential steps toward achieving more reliable diagnoses and optimizing patient care outcomes in cases of suspected neurosyphilis. The ability of clinical diagnosis and laboratory of neurosyphilis, especially in low-level hospitals need to be improved.

Treatment guidelines recommend high-dose intravenous penicillin G or intramuscular procaine penicillin G plus oral probenecid for neurosyphilis for 10–14 days to achieve sufficient penicillin concentrations in the CSF [13, 14]. In this study, two-thirds of patients of reported neurosyphilis cases received standard treatment. Notably, 87.3% of confirmed cases received standard therapy compared to 57.9% of suspected cases, resulting in significant improvement in psychotic symptoms as reflected by decreased BPRS scores, particularly in confirmed cases [18]. These results underscore the critical importance of accurate diagnosis and adherence to standard treatment protocols in managing neurosyphilis to improve both syphilis cure rates and associated mental health outcomes.

This study primarily focused on the prevalence and treatment of neurosyphilis in select neurological hospitals in Guangdong but had several limitations. It included only two secondary and two tertiary hospitals out of 21 cities, excluding primary psychiatric hospitals. Additionally, potential missed diagnoses of neurosyphilis in mental patients with syphilis and incomplete classification of syphilis stages due to limited clinical data were not analyzed.

The diagnosis and treatment of neurosyphilis in psychiatric hospitals in Guangdong, China, remains challenging. Only one-third of reported neurosyphilis cases were confirmed and two-thirds received standard therapy. Correct diagnosis and standard treatment is of clinical significance not only for curing syphilis but also for improving mental disorders caused by neurosyphilis. Enhancing diagnostic accuracy through greater use of CSF tests and implementing standardized treatment protocols are critical for effectively managing neurosyphilis and its associated mental health complications in psychiatric hospitals worldwide.

## 5. Conclusions

In Guangdong’s psychiatric field, given the need to manage syphilis and its associated mental health issues from neurosyphilis effectively, accurate diagnosis and standardized treatment are crucial. However, diagnosing and treating neurosyphilis in local psychiatric hospitals remain challenging, calling for enhanced clinical skills, updated tools, and strict protocol adherence to improve patient care and control the disease.

## Availability of Data and Materials

The datasets used and/or analyzed during the current study are available from the corresponding author upon reasonable request.
